# 2,3-Diphosphoglycerate and the Protective Effect of Pyruvate Kinase Deficiency against Malaria Infection—Exploring the Role of the Red Blood Cell Membrane

**DOI:** 10.3390/ijms24021336

**Published:** 2023-01-10

**Authors:** Maria Carvalho, Márcia M. Medeiros, Inês Morais, Catarina S. Lopes, Ana Balau, Nuno C. Santos, Filomena A. Carvalho, Ana Paula Arez

**Affiliations:** 1 Global Health and Tropical Medicine (GHTM), Instituto de Higiene e Medicina Tropical (IHMT), Universidade NOVA de Lisboa (UNL), 1349-008 Lisbon, Portugal; 2 Instituto de Medicina Molecular, Faculdade de Medicina, Universidade de Lisboa, 1649-028 Lisbon, Portugal

**Keywords:** malaria, 2,3-diphosphoglycerate, red blood cell, erythrocyte, morphology, membrane properties

## Abstract

Malaria remains a major world public health problem, contributing to poverty and inequality. It is urgent to find new efficacious tools with few adverse effects. Malaria has selected red blood cell (RBC) alterations linked to resistance against infection, and understanding the protective mechanisms involved may be useful for developing host-directed tools to control *Plasmodium* infection. Pyruvate kinase deficiency has been associated with resistance to malaria. Pyruvate kinase-deficient RBCs display an increased concentration of 2,3-diphosphoglycerate (2,3-DPG). We recently showed that 2,3-DPG impacts in vitro intraerythrocytic parasite growth, induces a shift of the metabolic profile of infected cells (iRBCs), making it closer to that of noninfected ones (niRBCs), and decreases the number of parasite progenies that invade new RBCs. As an increase of 2,3-DPG content may also have an adverse effect on RBC membrane and, consequently, on the parasite invasion, in this study, we explored modifications of the RBC morphology, biomechanical properties, and RBC membrane on *Plasmodium falciparum* in vitro cultures treated with 2,3-DPG, using atomic force microscopy (AFM)-based force spectroscopy and other experimental approaches. The presence of infection by *P. falciparum* significantly increased the rigidity of parasitized cells and influenced the morphology of RBCs, as parasitized cells showed a decrease of the area-to-volume ratio. The extracellular addition of 2,3-DPG also slightly affected the stiffness of niRBCs, making it more similar to that of infected cells. It also changed the niRBC height, making the cells appear more elongated. Moreover, 2,3-DPG treatment influenced the cell surface charge, becoming more negative in treated RBCs than in untreated ones. The results indicate that treatment with 2,3-DPG has only a mild effect on RBCs in comparison with the effect of the presence of the parasite on the host cell. 2,3-DPG is an endogenous host metabolite, which may, in the future, originate a new antimalarial tool with few adverse effects on noninfected cells.

## 1. Introduction

The most recent World Malaria Report estimates that malaria was responsible for over 240 million cases in 2021, from which approximately 620,000 resulted in deaths, representing a 12% increase relative to 2019 before the COVID-19 pandemic but maintaining a stable rate since 2020 [1]. Malaria is caused by parasites of the *Plasmodium* genus, of which *Plasmodium falciparum*, globally distributed, causes most cases and mortality worldwide.

In mammalian hosts, parasites go through an asymptomatic liver stage followed by an intraerythrocytic cycle in the blood, when malaria symptoms occur and during which the host cell is extensively remodeled by the parasite [2]. As malaria parasites and the human host have coexisted over millennia, alterations in host cell proteins may interfere with the normal survival of the parasites, potentially protecting the host from *Plasmodium* spp. infection and malaria disease. As such, there has been a selection for red blood cell (RBC) disorders related to host resistance to infection in populations from endemic areas for malaria [3].

Both RBCs and blood stage asexual parasites depend almost exclusively on the glucose in plasma as a source of energy for their metabolic activity and functions [4,5,6]. In glycolysis, the enzyme pyruvate kinase (PK; EC:2.7.1.40) catalyzes the conversion of phosphoenolpyruvate to pyruvate, with the production of one adenosine triphosphate (ATP) molecule [7]. PK deficiency (PKD) is an inherited enzymopathy that has been linked to reduced malaria susceptibility [8,9,10,11]. Malaria protection acquired from PKD was associated with accelerated senescence and phagocytosis of early-stage parasite infected RBCs, but the mechanism of protection is not yet clearly understood.

There is a decline in the levels of ATP inside PK-deficient RBCs, along with an increase in the concentration of the glycolysis intermediate metabolite 2,3-diphosphoglycerate (2,3-DPG) [7,8]. In mammalian RBCs, 2,3-DPG is synthesized by bisphosphoglycerate mutase (BPGM) in the Rapoport–Luebering shunt. It is present in RBCs at approximately 5 mM, and its main function is to regulate the affinity of hemoglobin to oxygen [4,12]. Increased 2,3-DPG levels inhibit glucose-6-phosphate dehydrogenase (G6PD) activity, and the antioxidant defense from glutathione becomes defective. An increased 2,3-DPG concentration also changes the cell membrane stability, as there is a disruption of the cytoskeletal–protein interactions, such as those of spectrin and actin, which leads to membrane instability and altered RBC deformability [7,13].

Although the malaria parasite possesses its own glycolytic enzymes, which are similar but structurally and biochemically different from those of the human host, it does not have an equivalent to BPGM. Thus, neither 2,3-DPG nor an equivalent molecule are synthesized by the malaria parasite [14]. The oxidative stress caused by the increase of 2,3-DPG concentration presumably makes the intraerythrocytic environment less hospitable to *Plasmodium* spp. and may be involved in the mechanism of protection against infection provided by PKD. In fact, Morais et al. [15] observed an impairment of *P. falciparum* growth in vitro under the effect of 2,3-DPG added to the culture medium. As 2,3-DPG affects membrane proteins, altering the host cell’s membrane, it may contribute to an increased RBC clearance, as well as also interfere with the parasite ability to infect new RBCs.

Knowing that PKD and the associated increase in 2,3-DPG content may have an effect on the host cell, we (1) studied the effect of the addition of 2,3-DPG on human RBCs by analyzing cell viability and changes on RBC morphological, biomechanical and membrane surface potential properties of non-parasitized and parasitized RBCs from in vitro *P. falciparum* cultures cultivated in the presence and absence of the compound and (2) evaluated if the changes in the RBC properties may compromise the invasion by the parasite.

To investigate the RBC morphological and biomechanical properties of non-parasitized and parasitized cells, we combined atomic force microscopy (AFM) with optical and fluorescence microscopy. Although AFM has been applied to study mechanical properties of tissues, cells, viruses and biological membranes, as well as proteins and other biomolecules, and has been used to study cancer, cardiovascular diseases, diabetes and infectious diseases, AFM studies regarding malaria-infected RBCs are still scarce [16,17,18]. In this study, AFM and optical microscopy enabled the detection, with high resolution, of morphological changes in the entire sample, as well as allowed the identification of infected RBCs in a mixture with noninfected RBCs.

## 2. Results

### 2.1. AFM Force Spectroscopy—Red Blood Cell Stiffness

**Figure 1:**
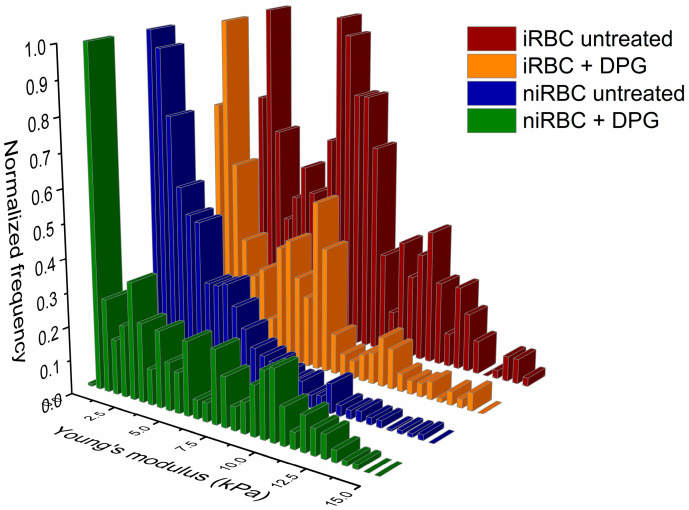
Histograms of RBC stiffness (Young’s modulus) measured by AFM force spectroscopy of infected and noninfected RBCs after 30 h of incubation with or without 2,3-DPG 8 mM. Parasites in infected samples were at the trophozoite stage. This figure includes data from three independent experiments. iRBC + DPG—infected RBCs treated with 2,3-DPG (*n* = 1573); iRBC untreated—untreated infected RBCs (*n* = 1126); niRBC + DPG—noninfected RBCs treated with 2,3-DPG (*n* = 1358); niRBC untreated—untreated noninfected RBCs (*n* = 1273). All descriptive statistics and *p*-values generated by Dunn’s multiple comparisons test are presented in Table S1.

Figure 1 shows that RBCs infected with *P. falciparum* (iRBCs) trophozoites, both treated with 2,3-DPG (Young’s modulus 5.12 ± 4.36 kPa, mean ± SD) and untreated (5.64 ± 3.85 kPa), are the most rigid cells (*p* < 0.0001). There is a significant increase in membrane rigidity of iRBCs in comparison with untreated noninfected RBCs (niRBCs) (3.33 ± 3.40 kPa, *p* < 0.0001). The latter are the most elastic samples. niRBCs become significantly more rigid when treated with 2,3-DPG (4.72 ± 3.89 kPa) than untreated niRBCs (*p* < 0.0001) but still have a lower Young’s modulus than infected cells.

**Figure 2:**
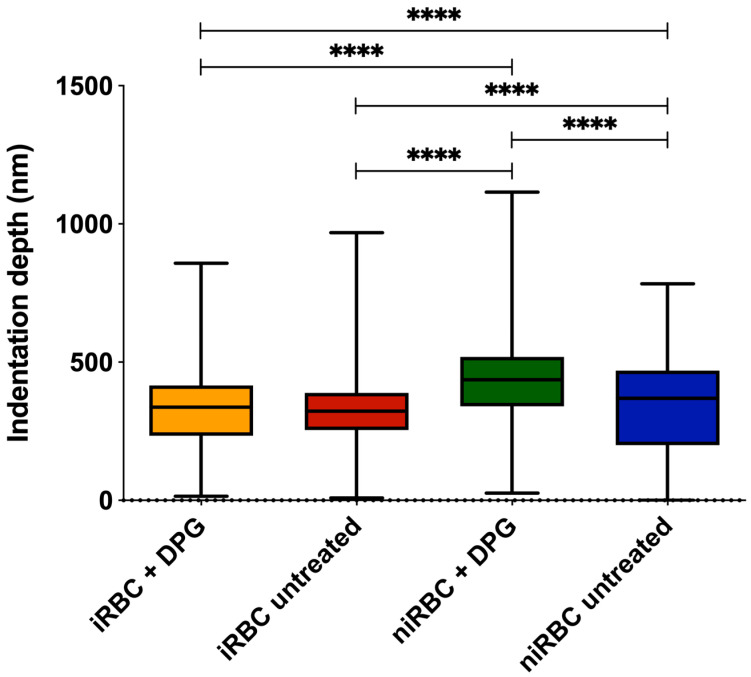
RBC indentation depth measured by AFM force spectroscopy of infected and noninfected RBCs after 30 h of incubation with or without 2,3-DPG 8 mM. Parasites in infected samples were at the trophozoite stage. This figure includes data from three independent experiments. iRBC + DPG—infected RBCs treated with 2,3-DPG (*n* = 1573); iRBC untreated—untreated infected RBCs (*n* = 1126); niRBC + DPG—noninfected RBCs treated with 2,3-DPG (*n* = 1436); niRBC untreated—untreated noninfected RBCs (*n* = 1358). *p*-values were generated by Dunn’s multiple comparisons test (**** *p* < 0.0001). Box plots: error bars represent minimum and maximum values; box higher and lower values represent the limits of the 1st and 3rd quartiles; lines represent mean for each sample group. All descriptive statistics and *p*-values are presented in Table S2.

The indentation depth results (Figure 2) showed that niRBCs treated with 2,3-DPG were the most deformable samples, with a mean membrane indentation of 409.6 ± 167 nm, significantly different from untreated niRBC (338 ± 174.6 nm). There were no statistically significant differences between iRBCs treated with 2,3-DPG or untreated (319.6 ± 149.6 nm vs. 318.2 ± 124.7 nm, respectively), being the cells less indented by the AFM probe or less deformable. 

### 2.2. AFM Imaging

Red blood cell morphology data was obtained by raster scanning the samples with the AFM probe, creating a topographic map of the cells. It was not possible to specifically select parasitized cells from cultures containing iRBCs (see Section 4.4.2 in Materials and Methods), and data concerning these samples considers both parasitized and non-parasitized cells. It was possible to observe specific patterns and significant morphological differences between infected and noninfected cultures.

**Figure 3:**
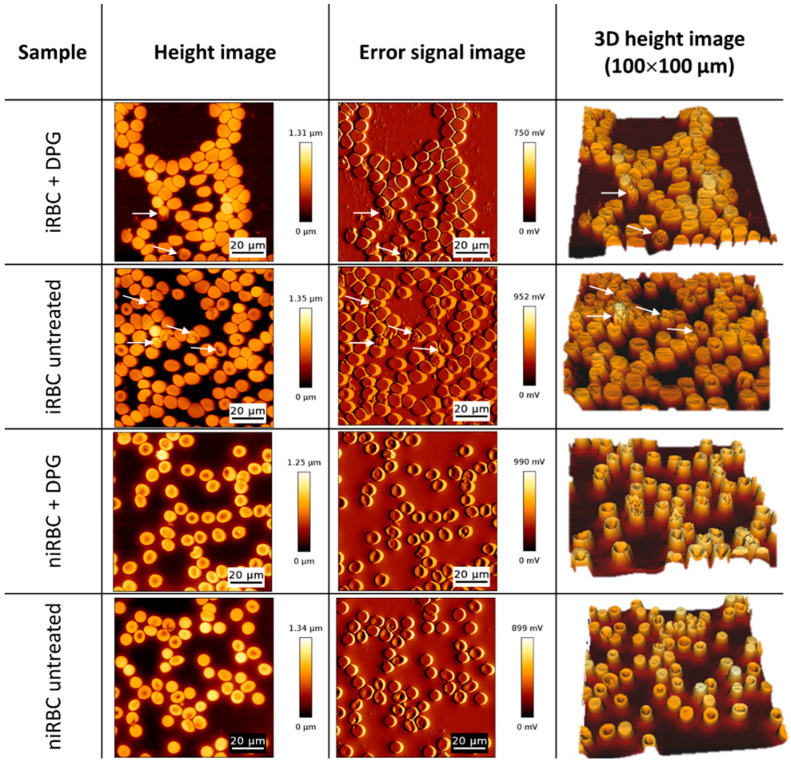
Atomic force microscopy images of cultures with infected RBCs (iRBC + DPG; iRBC untreated) and noninfected RBCs (niRBC + DPG; niRBC untreated) treated with 2,3-DPG and untreated, respectively. Height, error signal and “3D” images (100 µm × 100 µm). White arrows indicate trophozoite-infected RBCs.

**Figure 4:**
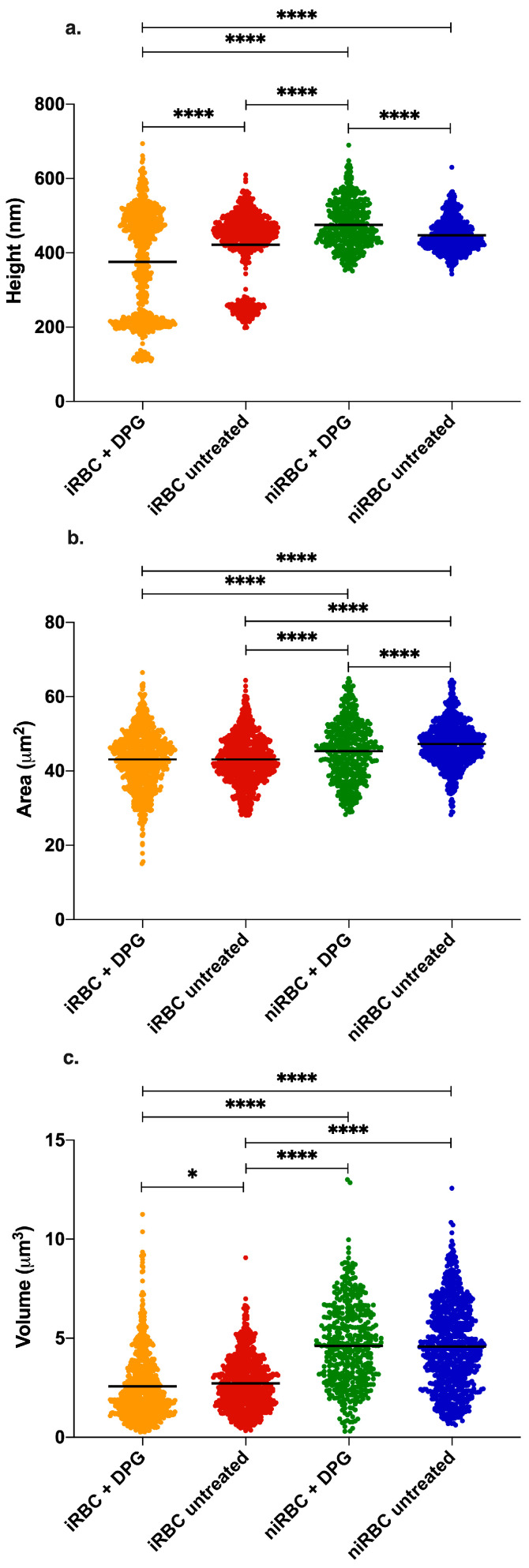
AFM imaging results of infected cultures and uninfected RBCs after 30 h incubation with or without 2,3-DPG 8 mM. (**a**) RBC height; (**b**) RBC area; (**c**) RBC volume. Parasites in infected samples were at the trophozoite stage. Each panel includes data from three independent experiments. iRBC + DPG—cultures with infected RBCs treated with 2,3-DPG (height *n* = 765; area *n* = 771; volume *n* = 766); iRBC untreated—cultures with infected RBCs untreated (height *n* = 741; area *n* = 734, volume *n* = 741); niRBC + DPG—noninfected RBCs treated with 2,3-DPG (height *n* = 432; area *n* = 429, volume *n* = 432); niRBC untreated—untreated noninfected RBCs (height *n* = 718; area *n* = 783; volume *n* = 812) (* *p* = 0.0444 and **** *p* < 0.0001; *p*-values were generated by Dunn’s multiple comparisons test). All descriptive statistics and *p*-values are presented in Tables S3–S5. The infection by *P. falciparum* had consistently more impact on the cell biomechanical properties and morphology than the presence of 2,3-DPG amongst all the AFM experiments, iRBC being more rigid and smaller than niRBC.

As AFM imaging was performed in air on cells deposited on glass slides, the values obtained for RBC height and volume are lower than the values found in the literature for RBCs in the buffer. Figure 3 shows AFM images illustrating the morphology results presented in Figure 4. Images from untreated niRBC cultures show RBC typical discoid, a biconcave shape (Figure 3, 4th row). As described in the cell morphology results, by comparing images from treated niRBCs (Figure 3, 3rd row) with those from cells in all other conditions studied (Figure 3, 1st, 2nd and 4th rows), it is possible to notice the increased height of RBCs measured by AFM. 

In the sample images depicted in Figure 3, 1st and 2nd rows, we may observe images of both treated and untreated iRBC cultures, respectively, where white arrows point to some infected cells. Differences in the cell area and volume between infected and noninfected cells are noticeable (Figure 3). Parasitized cells are generally smaller than surrounding RBCs, but, interestingly, the latter are also smaller than those from noninfected cultures. Additionally, AFM errors and 3D images from infected culture samples show the alterations in membrane roughness of parasitized cells more clearly than it is possible to observe in height images.

Figure 4a shows the morphological data of a RBC surface exposed after adhesion to a plane. Statistically significant differences between the RBC height values of all samples are seen. niRBC treated with 2,3-DPG presented the highest mean height values (476 ± 63 nm; mean ± standard deviation; *p* < 0.0001), while treated cultures with iRBC presented the lowest (376 ± 140 nm; *p* < 0.0001). Nonetheless, even with a low parasite density in the infected samples, the dot pattern in Figure 4a clearly shows that there are marked differences between the height of parasitized and non-parasitized cells among the iRBC samples (non-parasitized cells between 400 and 600 nm and parasitized cells with lower height values around 200–300 nm) when compared to both niRBC samples.

The untreated niRBC showed a significantly higher exposed surface area (47 ± 5.9 μm^2^; *p* < 0.0001) than all the other samples (Figure 4b), which hints that both the exposure to 2,3-DPG and the infection by *P. falciparum* may decrease this parameter. Figure 4c shows that both untreated and treated niRBC have the largest volumes (4.6 ± 2.1 μm^3^ and 4.6 ± 2.1 μm^3^, respectively) than both the untreated and treated cultures with iRBC (2.7 ± 1.3 μm^3^ and 2.6 ± 1.7 μm^3^, respectively). This steep decline in RBC volume is due to the presence of infected cells and may be caused by the parasite inside them. The presence of 2,3-DPG does not seem to have a significant effect on the volume of cells in the cultures with iRBC (*p* = 0.0444).

### 2.3. RBC Surface Charge Analysis

**Figure 5:**
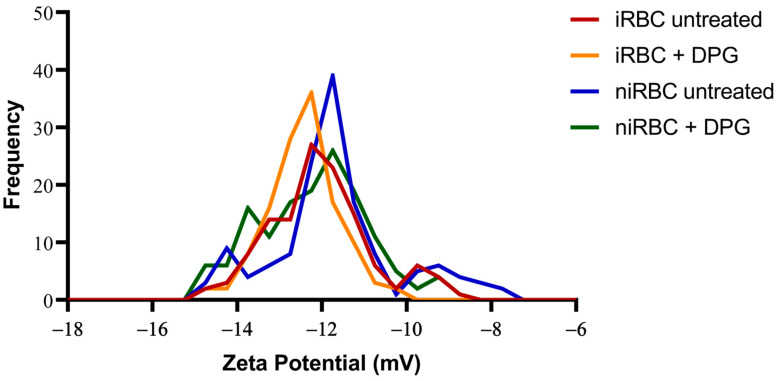
Histograms of the zeta potential of infected cultures and noninfected RBCs untreated or treated with 2,3-DPG for 30 h. Parasites in infected samples were at the trophozoite stage. This figure includes data from three independent experiments. iRBC + DPG—cultures with infected RBCs treated with 2,3-DPG (*n* = 124); iRBC untreated—cultures with infected RBCs untreated (*n* = 125); niRBC + DPG—noninfected RBCs treated with 2,3-DPG (*n* = 143); niRBC untreated—untreated noninfected RBCs (*n* = 139).

Similar to the AFM measurements, iRBC and niRBC cultures treated and untreated with 2,3-DPG at 8 mM were incubated for 30 h at standard conditions prior to the zeta potential measurements, which assess a cell’s surface charge. The zeta potential results from cultures with infected RBCs included parasitized and non-parasitized cells, with a parasite density of approximately 1%. Both iRBC cultures, treated or untreated (−12.52 ± 0.81 mV and −12.09 ± 1.22 mV, respectively) and treated niRBC cultures (−12.23 ± 1.303 mV), showed lower mean values of cell surface zeta potential when compared to untreated niRBCs, the samples with the highest mean value (−11.76 ± 1.48 mV; *p* < 0.001) (Figure 5). All descriptive statistics and *p*-values are presented in Table S6.

The presence of only a small percentage of cells infected with *P. falciparum* appeared to affect the whole culture and make the RBC membrane surfaces more negatively charged; 2,3-DPG also seemed to influence the membrane surface charge of both infected and noninfected samples, making them more negatively charged than their untreated counterparts.

### 2.4. Cell Viability and Re-Invasion Assays

niRBC treated with 2,3-DPG showed some alterations in RBC rigidity, membrane indentation, cellular height and zeta potential data. RBCs from these samples were less rigid, more indentable by the AFM probe and more elongated than the untreated cells. We asked if these alterations caused by the addition of 2,3-DPG to the culture medium of niRBC could affect the cell viability or if it could impair the parasite ability to reinvade a new niRBC previously submitted to the action of the compound.

**Figure 6:**
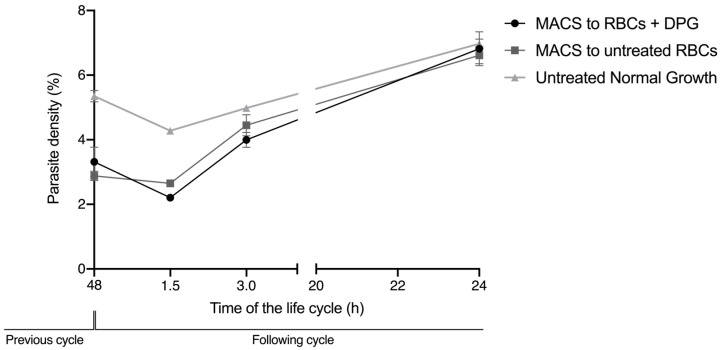
Total parasite density of infected RBCs during the reinvasion assay, measured by flow cytometry in at 0, 1.5, 3 and 24 h of the new development cycle. MACS to RBCs + DPG—iRBCs re-cultured in 2,3-DPG treated RBCs after magnetic enrichment of schizonts; MACS to untreated RBCs—iRBCs re-cultured in untreated RBCs after magnetic enrichment of schizonts; Untreated Normal Growth—iRBCs from cultures allowed to grow for a full developmental cycle under standard conditions. These data are representative of three independent experiments performed in triplicate. (MACS to RBCs + DPG—0 h, Mean ± SD = 3.31% ± 0.46; 1.5 h, Mean ± SD = 2.21% ± 0.09; 3 h, Mean ± SD = 4.00% ± 0.23; 24 h, Mean ± SD = 6.82% ± 0.53; MACS to untreated RBCs—0 h, Mean ± SD = 2.88% ± 0.15; 1.5 h, Mean ± SD = 2.65% ± 0.11; 3 h, Mean ± SD = 4.45% ± 0.33; 24 h, Mean ± SD = 6.61% ± 0.26; Untreated Normal Growth—0 h, Mean ± SD = 5.35% ± 0.18; 1.5 h, Mean ± SD = 4.28% ± 0.03; 3 h, Mean ± SD = 4.98% ± 0.09; 24 h, Mean ± SD = 6.97% ± 0.14).

The addition 2,3-DPG 8 mM did not reduce the viability of the RBCs, either infected or noninfected, as the cell viability was 100% for all samples. Regarding reinvasion assays, as depicted in Figure 6, the parasite density levels progressed similarly between the samples that were re-cultured after magnetic-activated cell sorting (MACS) of the mature parasites in both treated and untreated RBCs, which hints that the alterations observed in the membranes of treated niRBCs did not reduce the parasite’s ability to invade these cells. All cultures presented a regular escalation in parasite density levels in the new cycle. Regarding nonenriched cultures (untreated normal growth), the initial parasite densities were higher, because these did not undergo the selection of mature parasites, and ring-stage forms from the following cycle, which had already finished the previous developmental cycle prior to the magnetic enrichment time point, remained in the culture.

## 3. Discussion

We have been investigating how the glycolytic metabolite 2,3-DPG may be involved in the protective effect against malaria caused by PKD. Morais et al. [15] showed that, when 2,3-DPG 8 mM was added daily into a culture medium, there was a sharp reduction in parasite densities and a reduction in parasite progeny at the end of an intraerythrocytic developmental cycle. Furthermore, a shift of the metabolic profile of iRBC treated with 2,3-DPG, making it closer to that of niRBC, was observed. We conjectured if changes on RBC cytoskeleton or/and membrane properties caused by the compound could also contribute to these results by interfering with the *P. falciparum* invasion.

The presence of the parasite had consistently more impact on the cell biomechanical properties and morphology than the presence of 2,3-DPG. The extracellular addition of 2,3-DPG slightly affected the stiffness of niRBCs and niRBC heights, making the cells appear more elongated. 2,3-DPG treatment also influenced the membrane surface charge, as it is more negative in treated RBCs than in untreated ones.

These effects on the deformability of noninfected cells and on the RBC zeta potential were not sufficient to impair the efficient reinvasion of new RBCs upon exposure to 2,3-DPG. Thus, it cannot significantly contribute to the substantial decrease in the number of parasites that start the following cycle, as previously observed [15]. However, in the reinvasion assay, there was never a mixture with 2,3-DPG between the initial culture conditions before the magnetic selection of mature parasites, and the second condition in which the enriched parasites were re-cultured in previously treated RBC, by then washed and cultured in a medium without 2,3-DPG addition. This means that, when merozoites egress from the host cell and reinvade a new cell, the parasites have never been directly exposed to 2,3-DPG in the culture media. Knowing that the compound exerts some action in the membrane of RBCs, we cannot exclude that it may also interfere with the membrane of merozoites in this short time interval of exposure, which could further reduce the number of parasite capable of infecting new cells.

As previously said, 2,3-DPG displayed only a mild effect on the host cell properties compared to the effect of infection. iRBCs were consistently the most rigid and smaller cells with RBC membrane surfaces more negatively charged, and, once infected, no differences were seen between the treated and untreated cells.

During its growth, *P. falciparum* extensively remodels its host cell, not only exporting its own proteins into the RBC cytoplasm but also inserting proteins on the host cell membrane, changing the properties such as cytoadherence, permeability, cellular stiffness, rigidity and deformability [6,19,20,21]. In addition, from the trophozoite until late schizont stages right before RBC lysis, the solid parasite itself contributes to an increased overall rigidity of the host cell, mostly due to the enlarged digestive vacuole in the latter stages of the intraerythrocytic cycle [22].

Our data on iRBC stiffness agree with previous research on the effect of *P. falciparum* infection in RBC membranes. The lipid bilayer and the actin–spectrin network of RBCs are the main factors responsible for cell elasticity [23]. Previous studies based on AFM imaging have shown that the gradual increase in stiffness of *P. falciparum*-infected RBCs is accompanied by the destabilization of the membrane’s spectrin network [24]. Kwon et al. [17] used AFM to investigate the biomechanical properties of RBCs infected with the murine malaria parasite *Plasmodium berghei* (ANKA strain) and showed that RBCs infected with schizonts were stiffer than both those infected with early-stage parasites and noninfected RBCs, which is in accordance with our results. Furthermore, they were able to associate increased stiffness with additional changes in the membrane cytoskeleton: as the parasites matured, the amount of fibrillar actin in the RBC membrane increased. RBC deformability is essential for circulation in the microvasculature of the host in capillary vessels narrower than RBCs. A reduction in the host cell elasticity and deformability seems to increase parasite virulence by becoming stiffer and consequently less deformable; it is likely that iRBCs are retained in the circulation for a longer period, escaping splenic clearance [25]. RBCs infected by *P. falciparum* also lose their typical disc shape and the area-to-volume ratio decreases in comparison to noninfected cells [17]. As the parasite becomes more metabolically active, iRBCs show an increase in membrane permeability to extracellular solutes [26,27]. If a more permeable iRBC retained the same volume as a niRBC, an increased influx of extracellular solutes accompanied by an increase in parasite volume as it matured could theoretically bring the host cell closer to the ‘critical hemolytic volume’, resulting in premature iRBC lysis [28]. Mauritz et al. [28] developed a mathematical model, which shows that the excess of hemoglobin consumption [29] and subsequent elimination from the host cell via parasite-encoded transporters called New Permeability Pathways (NPP) is, in fact, a protective mechanism against early iRBC lysis, as infected cells are able to reduce their volume and surface area [28,30].

It has been observed both in vitro and in vivo that phosphatidylserine (PS) exposure is increased in *Plasmodium*-infected RBCs, particularly in mature developmental stages of the parasites [30]. The asymmetry of cellular membranes is characterized by differences on the lipids found on the inner and outer membrane monolayers, which confers different properties to either layer of the membrane that are required for varied cell functions [31,32]. The negatively charged PS is a membrane amino phospholipid of particular importance normally found in the inner monolayer of the membrane. These facts may provide some explanation for the membrane surface potential results of infected cultures, if the membrane charge of parasitized cells is altered by *P. falciparum* and the parasites could, in turn, exert an effect on the surrounding non-parasitized cells, therefore causing a more negatively charged cell surface in cultures containing iRBCs than in those of niRBCs. In fact, significant changes observed in the morphology and surface charge, measured in cultures containing both non-parasitized RBCs and 1% of infected RBCs, hinted that non-parasitized RBCs exposed to the presence of the parasite might be affected by the metabolism and release of toxic products from the 1% iRBCs, leading to additional effects, even if more subtle than those on the iRBCs. Furthermore, it has been shown that RBC incubation in high-concentration glucose solutions leads to the hyperpolarization of the cell membranes, thus making them more negatively charged, as well as leading to wrinkling of the cell surface [33]. Knowing that the glucose uptake is much higher in iRBCs than in niRBCs [6], this could also explain both the more negatively charged membranes of iRBCs and the wrinkling observed in the images obtained of both the treated and untreated iRBC samples.

However, the 2,3-DPG treatment also influenced the membrane surface charge, as it is more negative in treated RBCs than in untreated. At present, we cannot confirm if 2,3-DPG in extracellular media affects the phosphorylation of RBC skeletal proteins, which would impact the membrane stability and affect its viscoelastic properties [21]. However, we have shown that RBCs treated with 2,3-DPG at 8mM maintain 100% viability, so that these changes observed in the cell’s elastic properties and zeta potential are not impactful enough to compromise the cells’ normal activities nor efficient reinvasion by merozoites. Furthermore, it has been shown that RBC membrane reorganization and deep biomechanical transformations can be reversible [34].

Previous results hinted the involvement of the RBC-specific metabolite 2,3-DPG in the protective mechanism afforded by PKD. Morais et al. [15] showed that mature schizonts divide into a lower number of merozoites when cultured in the presence of the compound than those cultured at standard conditions, indicating that the parasites are not developing as efficiently. They also observed that there are no changes in the ATP levels inside the host cell and that the metabolomic profile of noninfected cells either treated or untreated did not differ significantly, while the profile of iRBCs treated with 2,3-DPG became more similar to the profile of niRBCs than to that of untreated iRBCs, which suggests a main effect of the addition of the compound on the parasite rather than on the host cell. However, they did not confirm whether the compound had a direct effect on the RBC morphology, biomechanical properties or RBC membrane. Our thorough analysis indicates that treatment with 2,3-DPG has only a mild effect on RBCs, when compared with the effect of the parasite on the host cell, which maintains its viability, further pointing to an impairing effect of the compound directly on the parasite rather than on the host RBC.

Increased 2,3-DPG levels also occur in other RBC disorders such as beta thalassemia, G6PD deficiency or sickle cell disease [35,36,37], due to the activation of glycolysis upstream to the pyruvate kinase. These disorders have already been associated with protection against malaria infection [38], and we cannot exclude that this metabolite may also be involved in the mechanisms underlying this protective effect. 2,3-DPG is an endogenous host metabolite, which may, in the future, be a new antimalarial tool with few adverse effects for noninfected cells.

## 4. Materials and Methods

### 4.1. Blood Donors

Healthy-type 0 RBCs were collected from adult volunteer donors (*n* = 5). To rule out blood variants that could bias the effect on parasite growth, a sample of whole blood from each donor was used for DNA extraction and subsequent molecular diagnosis of the polymorphisms most common in Portugal, linked to hemoglobinopathies and RBC enzymopathies associated with malaria protection (HBB—hemoglobin subunit beta, *pklr*—pyruvate kinase, liver and red blood cell and g6pd genes), as described by Morais et al. [15]. All donors were wild types for all studied genes.

All blood donors were clearly informed that participation in the study was voluntary and confidential and were made aware of the objectives of the work. Each participant signed an informed consent form before blood collection, and a numerical code was assigned to each donor to maintain confidentiality.

### 4.2. Plasmodium Falciparum In Vitro Cultures

*Plasmodium falciparum* 3D7 parasites (BEI Resources MRA-102) were maintained in RBCs at 5% hematocrit at 37 °C in a wet atmosphere with 5% CO_2_, accompanied by daily complete Gibco Roswell Park Memorial Institute 1640 Medium (cRPMI) changes based on a protocol adapted from Trager and Jensen [39]. Parasite growth was monitored daily through estimation of the parasite density in 20% Giemsa-stained (Giemsa′s Azur-eosin–methylene blue, Sigma-Aldrich, Darmstadt, Germany) thin blood smears by calculating the percentage of infected cells.

### 4.3. Sample Treatment with 2,3-Diphosphoglycerate

To study the effect of the compound on infected Red Blood Cells (iRBCs) and noninfected Red Blood Cells (niRBCs) in culture throughout the assays, solutions of 2,3-DPG 1.33 M were prepared in ultrapure water from which intermediate dilutions were done with cRPMI. The synthetic compound 2,3-DPG was added to the culture medium at a concentration of 8 mM. This concentration was chosen after the IC50 assays conducted by Morais et al. [15] using concentrations of 2,3-diphospho-D-glyceric acid pentasodium salt (Sigma-Aldrich, Darmstadt, Germany) above the physiological levels of endogenous 2,3-DPG, where it was observed that there was impairment of a complete parasite cycle of growth in vitro for 50% of parasites after 48 h of treatment with 2,3-DPG 8 mM added to the culture medium.

### 4.4. Atomic Force Microscopy Assays

AFM assays were conducted to evaluate the effect of *P. falciparum* infection and of 2,3-DPG on RBCs regarding cell morphology (height, area, and volume) and biomechanical properties such as stiffness and deformability. Prior to all assays, *P. falciparum* cultures were synchronized by treatment with a 5% (*w/v*) D-sorbitol solution (Sigma-Aldrich) [40] to obtain a predominantly ring-stage parasite culture up to 6 h after invasion, confirmed by optical microscopy of thin blood smears. Culture media from all samples (with or without 2,3-DPG) were changed daily. Infected samples were prepared with a parasitemia of approximately 1% in triplicate and in excess volume, so that replicates from the same original culture could be used in both imaging and force spectroscopy AFM experiments using a NanoWizard 4 atomic force microscope (JPK Instruments, Berlin, Germany) mounted on top of an Axiovert 200 fluorescence-inverted optical microscope (Carl Zeiss, Jena, Germany). RBCs were collected for analysis after 30 h of incubation with or without the compound. AFM analysis of iRBC was performed when the cells were infected with *P. falciparum* trophozoites, corresponding to a period of 30 to 36 h post-invasion and in the same period for niRBC. To do so, 400 μL of each biological replicate were incubated with SYBR Green (Thermo Fisher Scientific, Waltham, MA, USA) (0.001% (*v/v*) in phosphate-buffered saline (PBS)) for 30 min. After washing the samples, pellets were resuspended in 400 μL of PBS 1× and placed at 4 °C to stop parasite development until the moment of analysis. Sample analysis was performed in DPG/control pairs, so that the waiting time was the same for each pair of samples untreated and treated with 2,3-DPG.

#### 4.4.1. Force Spectroscopy for RBC Stiffness Analysis

To analyze the elastic properties of RBCs, AFM-based force spectroscopy measurements were carried out in an aqueous environment. iRBC and niRBC were diluted to 0.1% hematocrit on PBS pH 7.4 and allowed to adhere on clean poly-L-lysine-coated glass slides. RBCs were firmly attached to the glass slide after 20 min of deposition at room temperature, and non-adherent RBCs were removed by sequential washes with the buffer. To perform AFM elasticity experiments, erythrocytes need to be adhered to a surface for the cells to be immobilized during the force spectroscopy measurements, and adsorption onto surfaces precoated with poly-L-lysine (PLL) was employed. Poly-L-lysine is a polycation commonly adsorbed on surfaces for strong attachment to the negatively charged glycocalyx of cells, such as on RBCs [41,42], and this attachment does not compromise cell viability or the overall cell structure.

Nonfunctionalized OMCL TR-400-type silicon nitride tips (Olympus, Tokyo, Japan) were used, and all measurements were performed in PBS buffer, pH 7.4. Specific triangular cantilevers were used, with tip radii of ∼2 nm, nominal spring constant of 0.03 N/m and a cantilever frequency of 15 kHz. The applied force was adjusted and maintained at 500 pN. Data were collected at 2 µm/s and a z-displacement range of 2 µm for each force–distance cycle after positioning the cantilever on top of a single cell. 

iRBCs were optically identified on the microscope by SYBR Green fluorescence (0.001% *v/v* in PBS) before performing the AFM analysis. For each sample replicate (*n* = 3), approximately 100 different RBCs were measured, and 7 force–distance curves were collected on each of them. Thus, in total, approximately 300 RBCs were analyzed per condition. All measurements were performed on the top of the border’s rim of the RBC, as previously optimized, and not inside its concavity. Values of the AFM tip indentation depths into the RBCs were also obtained for a maximum applied force of 500 pN. After pressing the cells with the AFM tip, minimal adhesion between the tip and the RBC surface could occur. However, only the approach curve from the force–distance cycle was analyzed to extract the Young’s modulus data. Differences of RBC elasticity were evaluated by analyzing the approach curves of the force–distance curves acquired. Data were analyzed to obtain the RBCs’ Young’s modulus using JPK Image Processing software v. 6.0.55 (JPK Instruments, Berlin, Germany) by applying the Hertzian model [43].

#### 4.4.2. AFM Scanning Images for Morphology Analysis

Imaging of the RBC samples was performed in the contact mode with RBCs deposited on glass slides and allowed to air dry to achieve a higher resolution on the images of the surfaces of the cells. Oxidized-sharpened silicon tips (ACL, Applied NanoStructures, Inc., Mountain View, CA, USA) with a tip height of 15 µm, resonance frequency of approximately 190 kHz, spring constant of 58 N/m and a cantilever length of 225 µm, were used for the imaging. Imaging parameters were adjusted to minimize the force applied on the scanning of the topography of the cells. The scanning speed was optimized to 0.3 Hz, and the acquisition points were 512 × 512 pixels (100 × 100 µm^2^).

Unlike AFM-based force spectroscopy measurements, it was not possible to specifically select parasitized cells from cultures containing iRBCs (with a density of approximately 1%). On the measurement conditions, an AFM image takes approximately 30 min to be completed, and for each sample, we acquire four different images on each day of the experiment; if only infected RBCs were addressed, data from only 12–15 RBCs would be obtained. Viewing that this result could be complemented by the optical visualization of select parasite-infected RBCs, we scanned all the RBCs in the sample to have a higher number of cells on each acquired AFM image.

Imaging data were analyzed with JPK Image Processing software v. 6.0.55 (JPK Instruments, Berlin, Germany). The area, height and volume of imaged cells were quantified using MountainsSPIP software v.8.0 (Image Metrology, Hørsholm, Denmark).

### 4.5. RBC Zeta Potential

For the zeta potential analysis, RBC samples were prepared as described above for the AFM assays, collected after 30 h of incubation with or without 2,3-DPG 8 mM and resuspended in PBS pH 7.4. The cell surface zeta potential of all samples was measured in a cell suspension. As such, the iRBC samples contain both parasitized RBC (with a parasite density of approximately 1%) and niRBC. This means that the zeta potential values for cultures with iRBCs obtained in these experiments correspond to the total zeta potential of the samples, as opposed to that of the single parasitized RBC analysis performed using AFM-based force spectroscopy.

The samples in PBS were filtered using a syringe filter with a 0.45 μm pore size (Whatman, Florham Park, NJ, USA) to remove large scattering particles, which would bias the light scattering measurements. The RBCs’ suspension was then diluted to a 0.035% hematocrit in PBS. Using a 1 mL syringe, the sample was injected into folded capillary cells DTS 1070 (Malvern, UK). 

Measurements were conducted on a Malvern Zetasizer Nano ZS (Malvern, UK), equipped with a He-Ne laser (λ = 632.8 nm). Standard Operator Procedure measurements were predefined as the settings for all measurements. The RBC zeta potential analysis was performed following the methodology described by Carvalho et al. [44]. Before each measurement, the samples were preincubated for 10 min. The zeta potential of the RBCs was determined at 25 °C, from the mean of 15 measurements, with 60 runs each, with an applied potential of 30 V and an ionic strength of the PBS buffer of approximately 0.15 M (137 mM NaCl, 2.7 mM KCl, 10 mM Na_2_HPO_4_ and 1.8 mM KH_2_PO_4_).

### 4.6. Cell Viability Assay

To determine if the addition of 2,3-DPG 8 mM or the infection with *P. falciparum* reduced the number of viable cells, cell viability assays were performed for the cultures and for the niRBCs, both following 48h of incubation with or without 2,3-DPG 8 mM, according to the protocol in Strober [45]. Trypan Blue stains nonviable RBCs a dark blue color due to damage in the membranes. Cells were counted in a Neubauer chamber using an inverted microscope with a 20× magnification. Viable and nonviable cells were counted separately, and then, the percentage viability was calculated as follows:

Viable cell count per mL = number of live cells × dilution factor × 10^4^

Nonviable cell count per mL = number of dead cells × dilution factor × 10^4^

Percentage viability = number of viable cells/total number of cells counted × 100

### 4.7. Reinvasion Assay

We designed a reinvasion assay to assess whether merozoites that have egressed from untreated RBC were able to start a new intraerythrocytic cycle by reinvading cells that were treated with 2,3-DPG for 48 h prior to infection. Synchronized ring-stage *P. falciparum* cultures were allowed to grow for a full cycle and then divided into three new cultures at 5% hematocrit and 1% density of the ring parasites. niRBCs were also maintained in vitro with the culture medium treated with 2,3-DPG at 8 mM, and all four cultures were incubated under standard culture conditions with media changes every 24 h. At approximately 44 h of the parasitic cycle, schizonts from two of the infected cultures were magnetically enriched by magnetic-activated cell sorting (MACS) using LD columns attached to a QuadroMACS separator (Miltenyi Biotec, Bergisch Gladbach, Germany). Schizonts from the third remaining infected cultures were not enriched to allow the comparison of parasite density progress under normal growth conditions. 

After magnetic enrichment, four 96-well plates were prepared as follows: (1) triplicates (100 μL) from one enriched culture were cultivated in treated RBC, with a final 0.3% hematocrit and parasite density of approximately 1%; (2) triplicates (100 μL) from a second enriched culture were cultivated in untreated RBC with a final 0.3% hematocrit and parasite density of approximately 1%; and (3) triplicates (100 μL) from the nonenriched culture were cultivated with a final hematocrit of 0.3%.

Plates were incubated at 37 °C in a humidified atmosphere with 5% (*v/v*) CO_2_ for parasite reinvasion. Parasite densities were read by flow cytometry (Cytoflex, Beckman Coulter, Brea, CA, USA) at approximately 48 h, followed by readings at 1.5, 3 and 24 h of the following cycle. Before each plate reading, wells were incubated for 45 min under standard conditions with SYBR Green solution (0.001% *v/v* in PBS), then centrifuged, and the cells were washed and resuspended in the same volume of PBS. Three independent assays were performed in triplicate. Circa 100,000 RBCs were analyzed, and the parasite density was calculated using FlowJo v. 10 software (Tree Start Inc., Ashland, OR, USA).

### 4.8. Statistical Analysis

For the AFM and zeta potential data, normality tests were performed for all sets of data, followed by nonparametric Kruskal–Wallis tests and Dunn’s multiple comparisons of data from iRBC vs. niRBC untreated and treated with 2,3-DPG. For the zeta potential and AFM RBC stiffness data, the frequency distribution was also analyzed. Statistical analysis was performed using GraphPad Prism v. 5 and v. 8 (GraphPad Software), as well as OriginPro 9.0. For all tests, the statistical significance level was set at *p* < 0.05 unless otherwise stated.

## 5. Conclusions

Following the WHO’s 2021 update on the Global Technical Strategy for Malaria 2016–2030, there is an urgent need to pursue new tools in the path toward malaria elimination [46]. To paraphrase the former WHO’s Director of the Global Malaria Program, “research based on a problem-solving approach is essential, since simply expanding the tools that already existed in 2015 is not enough” (Pedro L. Alonso. Personal Communication [47]).

Interventions targeting host’s metabolic pathways, with few side effects and creating a less favorable environment to the parasite instead of affecting the parasite directly, are very promising and avoid the development of resistance to therapies, as the targets are not within or produced by the pathogen, especially considering how prone *Plasmodium* spp. are to developing resistance to antimalarials. By understanding the protective mechanisms of PK deficiency, or any other RBC disorders associated with malaria protection, these could be useful tools in the fight against malaria.

## Data Availability

The datasets generated and analyzed during the current study are available from the corresponding author upon reasonable request.

## Supplementary Materials

The following supporting information can be downloaded at: https://www.mdpi.com/article/10.3390/ijms24021336/s1.
