# Supplementary material for: 2,3-Diphosphoglycerate and the Protective Effect of Pyruvate Kinase Deficiency against Malaria Infection—Exploring the Role of the Red Blood Cell Membrane
Source: Int J Mol Sci. 2023 Jan 10;24(2):1336. doi: 10.3390/ijms24021336 (PMC9866842; doi:10.3390/ijms24021336)
Supplement: Supplementary file 1 [file ijms-24-01336-s001.zip › ijms-2136472-supplementary.pdf]

Table S1 - RBC stiffness (Young's modulus) measured by AFM force spectroscopy of infected and non-infected RBCs after 30 h of incubation with or without 2,3-DPG 8 mM. Results from statistical analysis by comparison between the studied groups.

**Dunn's Multiple Comparison Test**	**Mean rank diff.**	**Significant P < 0.05?**	**Summary**	**Adjusted P value**
niRBC untreated vs iRBC untreated	-567.5	Yes	***	<0.0001
niRBC untreated vs iRBC + DPG	-409.9	Yes	***	<0.0001
niRBC untreated vs niRBC + DPG	-285.6	Yes	***	<0.0001
iRBC untreated vs iRBC + DPG	157.7	Yes	*	0.0425
iRBC untreated vs niRBC + DPG	282.0	Yes	***	<0.0001
iRBC + DPG vs niRBC + DPG	124.3	No	ns	-
**Kruskal-Wallis test Descriptive statistics**	**niRBC untreated**	**niRBC + DPG**	**iRBC untreated**	**iRBC + DPG**
Median	2.1872	3.78086	5.2657	4.21844
Mean	3.3285	4.7232	5.64284	5.11759
Std. Deviation	3.39957	3.89117	3.84977	4.36006
Std. Error of Mean	0.0974894	0.150217	0.172512	0.191755

Data from three independent experiments. iRBC + DPG – infected RBCs treated with 2,3-DPG (n=1573); iRBC untreated – untreated infected RBCs (n=1126); niRBC + DPG – non-infected RBCs treated with 2,3-DPG (n=1358); niRBC untreated – untreated non-infected RBCs (n=1273).

Table S2 - RBC indentation depth measured by AFM force spectroscopy of infected and non-infected RBCs after 30 h of incubation with or without 2,3-DPG 8 mM. Results from statistical analysis by comparison between the studied groups.

**Dunn's Multiple Comparison Test**	**Mean rank diff.**	**Significant P < 0.05?**	**Summary**	**Adjusted P value**
niRBC untreated vs iRBC untreated	-401.0	Yes	****	<0.0001
niRBC untreated vs iRBC + DPG	-281.6	Yes	****	<0.0001
niRBC untreated vs niRBC + DPG	736.2	Yes	****	<0.0001
iRBC untreated vs iRBC + DPG	119.4	No	ns	0.3224
iRBC untreated vs niRBC + DPG	-1137	Yes	****	<0.0001
iRBC + DPG vs niRBC + DPG	-1018	Yes	****	-
**Kruskal-Wallis test Descriptive statistics**	**niRBC untreated**	**niRBC + DPG**	**iRBC untreated**	**iRBC + DPG**
Median	369.6	436.5	323.3	337.4
Mean	338.0	409.6	318.2	319.6
Std. Deviation	174.6	167.0	124.7	149.6
Std. Error of Mean	4.739	4.407	3.715	3.772

Data from three independent experiments. iRBC + DPG – infected RBCs treated with 2,3-DPG (n=1573); iRBC untreated – untreated infected RBCs (n=1126); niRBC + DPG – non-infected RBCs treated with 2,3-DPG (n=1436); niRBC untreated – untreated non-infected RBCs (n=1358).

Table S3 - AFM imaging results - RBC height of infected cultures and uninfected RBCs after 30 h incubation with or without 2,3-DPG 8 mM. Results from statistical analysis by comparison between the studied groups.

**Dunn's Multiple Comparison Test**	**Mean rank diff.**	**Significant P < 0.05?**	**Summary**	**Adjusted P value**
niRBC untreated vs iRBC untreated	2.3	No	ns	>0.99
niRBC untreated vs iRBC + DPG	-202	Yes	****	<0.0001
niRBC untreated vs niRBC + DPG	317	Yes	****	<0.0001
iRBC untreated vs iRBC + DPG	-204	Yes	****	<0.0001
iRBC untreated vs niRBC + DPG	-315	Yes	****	<0.0001
iRBC + DPG vs niRBC + DPG	-519	Yes	****	-<0.0001
**Kruskal-Wallis test Descriptive statistics**	**niRBC untreated**	**niRBC + DPG**	**iRBC untreated**	**iRBC + DPG**
Median	439	466	450	421
Mean	447	476	422	376
Std. Deviation	43	63	94	140
Std. Error of Mean	1.6	3.1	3.5	5.1

Data from three independent experiments. iRBC + DPG – cultures with infected RBCs treated with 2,3-DPG (n=7650); iRBC untreated – cultures with infected RBCs untreated (n=741); niRBC + DPG – non-infected RBCs treated with 2,3-DPG (n=41032); niRBC untreated – untreated non-infected RBCs (n=718).

Table S4 - AFM imaging results - RBC area of infected cultures and uninfected RBCs after 30 h incubation with or without 2,3-DPG 8 mM. Results from statistical analysis by comparison between the studied groups.

**Dunn's Multiple Comparison Test**	**Mean rank diff.**	**Significant P < 0.05?**	**Summary**	**Adjusted P value**
niRBC untreated vs iRBC untreated	-483	Yes	****	<0.0001
niRBC untreated vs iRBC + DPG	-238	Yes	****	<0.0001
niRBC untreated vs niRBC + DPG	-230	Yes	****	<0.0001
iRBC untreated vs iRBC + DPG	45	No	ns	>0.9999
iRBC untreated vs niRBC + DPG	-253	Yes	****	<0.0001
iRBC + DPG vs niRBC + DPG	-208	Yes	****	-<0.0001
**Kruskal-Wallis test Descriptive statistics**	**niRBC untreated**	**niRBC + DPG**	**iRBC untreated**	**iRBC + DPG**
Median	47	46	43	44
Mean	47	45	43	43
Std. Deviation	5.9	7.8	6.6	7.7
Std. Error of Mean	0.21	0.38	0.24	0.28

Data from three independent experiments. iRBC + DPG – cultures with infected RBCs treated with 2,3-DPG (n=771); iRBC untreated – cultures with infected RBCs untreated (n=734); niRBC + DPG – non-infected RBCs treated with 2,3-DPG (n=429); niRBC untreated – untreated non-infected RBCs (n=783).

Table S5 - AFM imaging results - RBC volume of infected cultures and uninfected RBCs after 30 h incubation with or without 2,3-DPG 8 mM. Results from statistical analysis by comparison between the studied groups.

**Dunn's Multiple Comparison Test**	**Mean rank diff.**	**Significant P < 0.05?**	**Summary**	**Adjusted P value**
niRBC untreated vs iRBC untreated	-679	Yes	****	<0.0001
niRBC untreated vs iRBC + DPG	-794	Yes	****	<0.0001
niRBC untreated vs niRBC + DPG	27	No	ns	>0.9999
iRBC untreated vs iRBC + DPG	-116	Yes	*	0.0277
iRBC untreated vs niRBC + DPG	-706	Yes	****	<0.0001
iRBC + DPG vs niRBC + DPG	-822	Yes	****	-<0.0001
**Kruskal-Wallis test Descriptive statistics**	**niRBC untreated**	**niRBC + DPG**	**iRBC untreated**	**iRBC + DPG**
Median	4.5	4.5	2.6	2.1
Mean	4.6	4.6	2.7	2.6
Std. Deviation	2.1	2.1	1.3	1.7
Std. Error of Mean	0.075	0.099	0.049	0.062

Data from three independent experiments. iRBC + DPG – cultures with infected RBCs treated with 2,3-DPG (n=73966); iRBC untreated – cultures with infected RBCs untreated (n=741); niRBC + DPG – non-infected RBCs treated with 2,3-DPG (n=432); niRBC untreated – untreated non-infected RBCs (n=812).

Table S6 - Zeta potential of infected cultures and non-infected RBCs, untreated or treated with 2,3-DPG for 30 h. Results from statistical analysis by comparison between the studied groups.

**Dunn's Multiple Comparison Test**	**Mean rank diff.**	**Significant P < 0.05?**	**Summary**	**Adjusted P value**
niRBC untreated vs iRBC untreated	-38.41	No	ns	0.25
niRBC untreated vs iRBC + DPG	-98.84	Yes	***	<0.001
niRBC untreated vs niRBC + DPG	48.02	No	ns	0.05
iRBC untreated vs iRBC + DPG	60.43	Yes	*	0.01
iRBC untreated vs niRBC + DPG	09.62	No	ns	>0.99
iRBC + DPG vs niRBC + DPG	-50.81	Yes	*	0.04
**Kruskal-Wallis test Descriptive statistics**	**niRBC untreated**	**niRBC + DPG**	**iRBC untreated**	**iRBC + DPG**
Median	-11.80	-12.10	-12.10	-12.50
Mean	-11.76	-12.23	-12.09	-12.52
Std. Deviation	1.482	1.303	1.220	0.814
Std. Error of Mean	0.1257	0.1089	0.1091	0.07307
Gaussian peaks ± Std. Deviation	-11.82 ±0.03	-12.15 ±0.06	-12.15 ±0.04	-12.43 ±0.02

Data from three independent experiments. iRBC + DPG – cultures with infected RBCs treated with 2,3-DPG (n=124); iRBC untreated – cultures with infected RBCs un-treated (n=125); niRBC + DPG – non-infected RBCs treated with 2,3-DPG (n=143); niRBC untreated – untreated non-infected RBCs (n=139).
